# Supplementary material for: Complexome profiling of the Chlamydomonas psb28 mutant reveals TEF5 as an early PSII assembly factor
Source: Plant Cell. 2025 Mar 17;37(6):koaf055. doi: 10.1093/plcell/koaf055 (PMC12164590; doi:10.1093/plcell/koaf055)
Supplement: koaf055_Supplementary_Data [file koaf055_supplementary_data.zip › psb28_interactive.zip!/plots/intensity/qualityPlots/peptides/_Cre16.g696000.t1.2.html]

### Cre16.g696000.t1.2

Trivial name:   
SeqCoverage [median]: 25.70  
Mass: 156.04 kDa  
  
 

| Sequence | MissedCleavage | Mass [Da] | wtIntensity | muIntensity | Score |
| --- | --- | --- | --- | --- | --- |
| AAAAPGKAPAPGGGAAGYR | 1 | 1609.83 | 1235.47 | 942.37 | 74.14 |
| AAAAPGKAPAPGGGAAGYRLER | 2 | 2008.06 | 1056.89 | 6404.63 | 65.47 |
| ALAAEVGLNATMADPAVAAVR | 0 | 2010.06 | 537.18 | 0.00 | 44.79 |
| APAPGGGAAGYRLER | 1 | 1441.74 | 740.44 | 1252.79 | 41.09 |
| AQLAEAVEAR | 0 | 1056.56 | 8865.62 | 6409.51 | 111.46 |
| ASDAGLAAATADAIAK | 0 | 1415.73 | 4107.85 | 8684.96 | 214.81 |
| AVTGLVDR | 0 | 829.47 | 3815.16 | 9996.41 | 121.99 |
| EAVDKLDPVAGDGAVQHSAIK | 1 | 2119.09 | 2237.30 | 2762.75 | 44.24 |
| GQAALVTLGGATTEAGELR | 0 | 1813.95 | 1529.03 | 3919.95 | 127.32 |
| GVEQEAR | 0 | 787.38 | 755.14 | 0.00 | 86.47 |
| IKLSEDDAAR | 1 | 1116.58 | 5457.20 | 4169.63 | 87.57 |
| LAPADKLPSR | 1 | 1066.61 | 7697.37 | 11371.44 | 93.56 |
| LAVTAPPAAK | 0 | 937.56 | 1694.23 | 1109.15 | 70.92 |
| LEDALGALR | 0 | 956.53 | 1287.60 | 451.62 | 75.74 |
| LGGMVSALGSQPAK | 0 | 1314.70 | 1420.13 | 1735.14 | 120.77 |
| LLALESLLR | 0 | 1026.64 | 2233.51 | 7067.96 | 105.57 |
| LSQVSDEGER | 0 | 1118.52 | 1316.73 | 0.00 | 94.69 |
| LSQVSDEGERAK | 1 | 1317.65 | 5874.05 | 4445.09 | 90.73 |
| LTADIGLPSAK | 0 | 1084.61 | 4175.41 | 3937.12 | 160.36 |
| LTEAEAAVR | 0 | 958.51 | 10454.98 | 13252.03 | 157.68 |
| NLQVLK | 0 | 713.44 | 1069.44 | 2536.86 | 131.65 |
| SQPGSGEVFLALR | 0 | 1359.71 | 1084.58 | 0.00 | 86.01 |
| TAAADAVNSEADAAVAAER | 0 | 1801.84 | 6070.34 | 5765.22 | 201.30 |
| TASPTSAVDAMLLQQTR | 0 | 1788.90 | 1106.06 | 1206.61 | 84.86 |
| TLGEVVSQLGPIGGSVDAR | 0 | 1853.98 | 0.00 | 2883.25 | 60.53 |
| TRLTEAEAAVR | 1 | 1215.66 | 352.85 | 323.94 | 65.84 |
| TSQAAGLK | 0 | 774.42 | 3125.48 | 2181.49 | 82.72 |
| TTAPNNAIASLK | 0 | 1199.65 | 2444.11 | 3876.46 | 113.93 |
| TTLSAPLSLEAAAPAVAAQK | 0 | 1909.05 | 6029.36 | 9164.19 | 109.00 |
| TTVFGLK | 0 | 764.44 | 893.96 | 0.00 | 111.69 |
| TTVFGLKVER | 1 | 1148.66 | 448.69 | 827.03 | 41.54 |
| VAEIEAAAAK | 0 | 971.53 | 12440.72 | 13594.86 | 149.72 |
| VAVPGVQTEGTSALPELYSR | 0 | 2073.07 | 1595.14 | 5427.11 | 88.26 |
|  |  |  | 103152.0 | 135699.6 |  |
